# Follow *the* science? On the marginal role of the social sciences in the COVID-19 pandemic

**DOI:** 10.1007/s13194-021-00416-y

**Published:** 2021-10-22

**Authors:** Simon Lohse, Stefano Canali

**Affiliations:** 1 Institute for History of Medicine and Science Studies, University of Lübeck, Lübeck, Germany; 2 Centre for Ethics and Law in the Life Sciences, Leibniz University Hannover, Hannover, Germany; 3 African Centre for Epistemology and Philosophy of Science, University of Johannesburg, Johannesburg, South Africa; 4 Department of Electronics, Information and Bioengineering and META - Social Sciences and Humanities for Science and Technology, Politecnico Di Milano, Milan, Italy

**Keywords:** Public health, Scientific expertise, Pluralism, Interdisciplinarity, Evidence-based policy

## Abstract

In this paper, we use the case of the COVID-19 pandemic in Europe to address the question of what kind of knowledge we should incorporate into public health policy. We show that policy-making during the COVID-19 pandemic has been biomedicine-centric in that its evidential basis marginalised input from non-biomedical disciplines. We then argue that in particular the social sciences could contribute essential expertise and evidence to public health policy in times of biomedical emergencies and that we should thus strive for a tighter integration of the social sciences in future evidence-based policy-making. This demand faces challenges on different levels, which we identify and discuss as potential inhibitors for a more pluralistic evidential basis.

## Introduction

Policy decisions have been influenced by scientific evidence since the nineteenth century at least. However, the idea of placing scientific evidence at the centre of policy has more recent origins and has only been promoted as ‘evidence-based policy’ in the last few decades. Beginning in the UK in the 1990s, evidence-based policy was advocated in several important policy areas – such as social and environmental policy – and spread to many other countries in the world (Nutley et al., 2010). Today, policy-making relies on scientific expertise, knowledge, and evidence to an extent that it has never done before. In this paper we focus on a specific policy area where this strong dependency on scientific evidence has been highlighted to an extraordinary degree by the COVID-19 pandemic: public health. Our motivational starting point for this paper is the observation that public health measures and strategies meant to deal with the COVID-19 pandemic seemed to be predominantly informed by the biomedical sciences, in particular epidemiology.^1^ Biomedicine was in the epistemic centre of policy-making while other potentially relevant sciences, in particular the social sciences, played only a marginal role. We are not the first to note this: consider this critical statement by Mark Woolhouse, a professor of infectious disease epidemiology at the University of Edinburgh and member of SAGE in the UK (Scientific Advisory Group for Emergencies), that appeared in *The Guardian* in April 2020:

“I do think scientific advice is driven far too much by epidemiology – and I’m an epidemiologist. What we’re not talking about in the same formal, quantitative way are the economic costs, the social costs, the psychological costs of being under lockdown. […] I understand that the government is being advised by economists, psychiatrists and others, but we’re not seeing what that science is telling them” (Devlin & Boseley, 2020).

This critique of health policy in relation to the COVID-19 pandemic raises a number of questions: To what extent does the diagnosis of biomedicine-centric governmental response actually stand up to scrutiny? What does it mean to criticise the response to the pandemic for its lack of inclusion of other sciences (in the same formal way)? Would we actually need to integrate other areas of knowledge into public health policy? If so, how can we integrate social and biomedical sciences in particular?

In this paper, we discuss these questions by analysing science-policy relations in the COVID-19 context and explore the governmental response to the pandemic through the lens of ‘philosophy of science in practice’. Specifically, we follow this approach by focusing on the processes and activities, rather than (solely) on the products of scientific research, as the main units of our philosophical analysis (Chang, 2014). We believe that this approach is particularly suited for exploring the unfolding COVID-19 pandemic, as it allows for a timely consideration of ongoing scientific developments and their relation with policy-making. As a consequence of this approach we do not aim to build a final analysis of policy-making during the pandemic.^2^ Rather, we want to highlight and analyse certain aspects of evidence-based policy-making in the face of the COVID-19 pandemic that will help us to uncover deeper conceptual, epistemological, and methodological aspects of evidence-based policy. Our analysis will focus on developments in Europe, which has been one of the earliest areas to be affected by COVID-19 and has been a hotspot of the pandemic. Following a bottom-up approach, we get to the more conceptual and normative level of our analysis by treating examples from the European context as instantiations of the phenomena we intend to uncover and assess (see Morgan, 2012). Our approach also follows Lohse et al. (2020) by paying special attention to the entanglement of epistemic aspects and ethical, legal, and social issues of evidence-based policy.

We think the case of the COVID-19 pandemic is interesting in itself but can also be used to understand deeper epistemic issues in public health policy that “follows the science” (a claim made by several European governments) – in particular in relation to pluralism and knowledge integration. The core normative problem underlying these issues has not received much attention in the literature on evidence-based policy. Questions about the right kind of evidence and expertise are primarily discussed in terms of causal evidence, evidence hierarchies, and generalisability (Cartwright & Hardie, 2012; Cartwright & Stegenga, 2011). We believe, however, that health policy-making during the COVID-19 pandemic has drawn attention to yet another aspect of evidence-based policy: We need to think more about diversity of evidence and expertise from different sources and about integrating different scientific fields.

In discussing evidence-based policy in the context of COVID-19, we should note that policy-making never really follows science in a very strict sense, that it is always also a matter of power, politics, regulatory constraints, bureaucracy, and the “survival of the ideas that fit” (Stevens, 2007) on which policy-makers actually act. Many public health strategies and changes in COVID-19-related policy in late 2020 (and early 2021) in European countries can be used to illustrate this claim. We also agree with Jasanoff that “the scientific story presented to the public” although influenced by scientific advice “is finally a creation of the political process” (Jasanoff, 1987: 197f). However, this does not mean that we should give up on the idea of evidence-based policy or that normative reflections on pluralism in evidence-based policy are useless. Although policy is not “driven” or “determined” by science, scientific knowledge does have an influence on policy-making – in an indirect, transformed way, nested in political processes. Thus we need to reflect on normative issues that bear on the kinds of evidence that *should* be used for policy-making. Without this, we can hardly know in which ways we should improve science-oriented policy-making.

The paper is structured as follows. We start by discussing three main stages of COVID-19 policy-making, putting a special focus on science-policy relations and the reception of scientific expertise (Section 2). It will become clear that the biomedical sciences, in particular epidemiology, were indeed in the epistemic centre of all these stages. We will show that this holds true not only for the beginning of the pandemic, when authorities needed to act fast in view of a potentially severe health threat to the population, but also at a later stage of the response to the pandemic. Section 3 presents reasons for why this is problematic by highlighting elements that the social sciences could bring to the table to improve the understanding and management of pandemics. Based on this, we claim that we should strive for a tighter integration of the social sciences in future evidence-based public health policy. We then turn to key challenges to the realisation of this demand (Section 4), discussing conceptual issues in relation to the notion of ‘public health’, epistemological differences between biomedicine and the social sciences, and methodological problems in selecting, gathering and integrating data on social phenomena for policy-making. Section 5 concludes by drawing out normative implications of our analysis.

## Development stages of the pandemic in Europe

How did governmental bodies respond to the COVID-19 pandemic in Europe? This is a question to which there are different answers, depending on which aspects of COVID-19 policy one considers. In the context of this paper, we are particularly interested in the disciplinary and evidential basis of policy. We discuss several examples from continental Europe, in particular Italy and Germany (the countries we are based at), in order to expand the focus beyond often-discussed cases from the US and the UK. We are not aiming for a detailed historical account of events, but we want to highlight a number of noteworthy features in the relations between science and policy and the ways in which scientific expertise was requested and used on the basis of various examples. What emerges as a result of our analysis is that, throughout the pandemic, European governments and policy bodies predominantly relied on the biomedical sciences, experts in biomedicine and biomedical evidence, to guide their policy-making, while other disciplines played only a marginal role. With this we do not mean that non-biomedical issues were not considered at all by policy-makers. For instance, considerations regarding childhood education, the labour market, and funding aimed at the economic recovery of European countries after the pandemic have constantly been an important element of pandemic policy in Europe. Yet for the most part these and other policy decisions were considerably less based on evidence and expertise from the social sciences.

We discuss three main stages of pandemic policy: preparation, emergency, and long-term.^3^ The reasoning for using this typology in our analysis is twofold. In the policy literature, most paradigms and pathways for pandemic response utilise a distinction between public health strategies aimed at preparedness, emergency, and long-term as the three main areas of focus (Allen et al., 2020). The classification enables us to highlight how some of the phenomena that we want to discuss in the rest of the article were specific to one of the stages and how others have played an important role throughout the response to COVID-19. At the same time, this is a way for us to specify our analysis and break it down to individual cases and examples at different stages of policy. This allows us to analyse the temporal development of pandemic policy in relation to the timeline of COVID-19 outbreaks without restricting our claims to individual waves. Although these three stages are mostly subsequential, throughout the COVID-19 pandemic we have seen that for example emergency interventions were implemented several times and preparedness was a debated issue after the initial outbreaks and later on.

### Preparing for the emerging pandemic

Between the end of 2019 and the beginning of 2020, policy makers and disease control agencies in Europe started to monitor the development of the new SARS-CoV-2 Coronavirus that had its initial outbreak in China in December 2019. While individual countries are primarily responsible for public health and medical care, and EU institutions can only complement national policies, at this stage, many initiatives were initially led by the EU commission. Dedicated response teams were only set up in March though, with the establishment of a political team led by the EU Commission, an advisory panel composed of epidemiologists and virologists from different European countries and a communication support system for knowledge exchange between healthcare professionals on COVID-19.^4^

In the meantime, in late February 2020 a cluster of cases was detected in the north of Italy, in the regions of Lombardy, Veneto, and Emilia-Romagna. In late February, Italy was the worst-hit country in the EU in terms of the number of infections and deaths by COVID-19 (Remuzzi & Remuzzi, 2020). Regional and city authorities started to implement initial policy measures, which included local and restricted closing of schools and curfews for bars and cafes. The national government started to rely on the newly established crisis response team in early February (*Comitato Tecnico-Scientifico*, CTS), which has acted as a consultant for government policy throughout the pandemic (Pistoi, 2021).

The formation of expert and crisis teams is a crucial element of preparing for biomedical emergencies, which is why we are focusing on this specific element of the preparation stage in this section. This element is also a window into the choices and assumptions about the types of expertise and evidence that are considered necessary for dealing with an emergency. Right from these first steps, one of the points we want to emphasise was evident: while observatory and preparatory steps were led by political bodies at national and international levels, institutions largely relied on biomedical experts. For example, the Italian CTS is composed of biomedical experts, including medical doctors, epidemiologists, health professionals, and head physicians of some of the largest hospitals in the country, and members of the Ministry of Health.^5^ Similar steps were taken by other European governments in February and March. For instance, in the UK the Scientific Advisory Group for Emergencies (SAGE) was activated for the study and preparation of COVID-19 in late January. In Germany, local governments started to establish response teams in late February, in coordination with experts based at the Robert Koch Institute (RKI) and the Federal Ministry of Health. The RKI is the national public health institute in Germany, which relies on biomedical scientists based at various universities in Germany and works on the basis of specific sub-groups and committees on biomedical issues. A similar institution has been responsible for the COVID-19 crisis in Spain, where the government has relied on the Coordination Centre for Health Alerts and Emergencies based at the Ministry of Health. In France, the government set up its special experts team on COVID-19 (*Conseil scientifique Covid-19*), under the coordination of the ministry of health, comprising environmental and infectious disease epidemiologists, disease modellers, virologists, and bioinformaticians.

As we will see, these choices made at the preparation stage remained central beyond immediate emergencies, and this has been the case for most expert teams in Europe, which have continued to advise governments on policy during the summer and winter of 2020, sometimes in conjunction with other teams advising on more specific topics (Jasanoff et al., 2021).

### Emergency: crisis responses in Europe

In Spring 2020 two main events signalled the transition from the preparation to the emergency stage of the pandemic. In early March, the number of COVID-19 infections and deaths connected to the pandemic in the northern Italy region of Lombardy started to rise very significantly (Goumenou et al., 2020). The Italian government, on the basis of these developments and the coordination with the CTS, applied a ‘lockdown’ in Lombardy, which included the closure of all shops and activities considered non-essential and became nation-wide in mid-March.^6^ Around the same time, the COVID-19 Response Team based at Imperial College London published a modelling study that developed a number of different (conditional) scenarios for the development of the pandemic in the UK and the US (Ferguson et al., 2020). The study crucially predicted that, in case of no action by governments with decisive measures to suppress the spreading of the virus, many countries would see an uncontrolled development of the disease and a crisis of healthcare systems, with a consequently extremely high number of deaths.

The Lombardy case and the Imperial College Model shifted the focus of policy-makers and prompted governments to take swift actions, which were mostly informed by biomedical evidence and expertise (Adam, 2020). For instance, the actions taken in Italy were presented and justified on the basis of reproduction numbers of the SARS-CoV-2 Coronavirus that measures the average number of people that a person infected with the virus infect. In the epidemiology of infectious disease and epidemics, reproduction numbers are key figures to understand the spreading of disease, as they depend both on biological features of a virus and the development stage of an epidemic. The use of reproduction numbers continued to be central for justifying policy measures in subsequent emergency situations in Europe, in particular during the second and third waves of 2020 and early 2021. In addition, governments started to rely on biomedical indicators such as incidence, hospitalisation rate, and number of available beds in ICUs (intensive care units) to assess local developments and deploy more specific mitigation measures (see e.g. the colour-based systems applied in Germany and Italy and the tier system used in the UK).

Predictive (and also backwards directed) modelling has remained a central source of guidance for European governments (Chin et al., 2021). In particular sophisticated agent-based models, like the Imperial College model, which simulate interactions between susceptible and infected agents in a population over a certain time interval, have been very influential modelling tools (next to equation-based models and curve-fitting approaches, see Fuller, 2021). Although this type of model uses census data on some social/demographic characteristics (e.g. household distribution and population age characteristics) to calibrate several parameters, it is crucially based on a biomedical rationale and aims at a projection of the effects of the spreading of the disease and of different mitigation strategies on the number of deaths, infection rates, hospitalisations, and the healthcare system.

The centrality of the biomedical lens and focus at this stage of the pandemic has also been evident in the broader public sphere, in particular in the media and on social media platforms. Media discussions started to heavily use biomedical data and concepts, including the reproduction number R, and relied on biomedical experts, especially from virology and epidemiology. This has in turn influenced policy-making. A striking example for this claim is the highly influential *Medium* article “Coronavirus: The Hammer and the Dance” (Pueyo, 2020), which outlined a COVID-19 strategy that has even been discussed in a 2020 white paper by the federal government in Germany.^7^

### Long-term: policy-making in light of COVID-19

A last stage of pandemic policy-making we want to discuss is long-term planning. In Europe, this stage has been most evident in spring/summer 2020, sometimes described as the ‘new normal’. In this context, many governments tried to apply more targeted measures and took a step back from large-scale interventions. Quarantine periods were introduced for travellers coming from areas deemed as risky on the basis of infection and reproduction rates of COVID-19. Since spring 2020, the German RKI has kept a running list of risk areas, which is updated weekly to include areas, cities, or entire countries from which travelling entails the need for a negative test or a quarantine period. The targeted approach also includes COVID-19 policy regimes that have emerged in several European countries at this stage of the pandemic, i.e. tier systems where specific regions of countries are assigned different and more or less severe levels of restrictions and interventions (e.g. school and university closures, economic lockdown measures, and contact restrictions), depending on the infection levels in the area. These and similar regulations have changed continuously and substantially, as governments have tweaked the number of days that constitute a quarantine, the type of test required at entry and the time at which it should be conducted – the variability and instability of policy interventions has been a general feature of long-term COVID-19 management.

Governments clearly continued to rely on expertise and evidence mostly coming from the biomedical sciences at this stage. For instance, the development of smartphone applications in the summer of 2020 has been discussed in terms of epidemiological considerations and approaches to tracking and tracing (Leonelli, 2021a). Biomedical concerns were at the basis of investments aimed at increasing the number of ICU beds and infrastructure and testing rates: this was particularly evident in countries such as Spain and Italy, which have a low density of ICUs compared with countries such as Germany, as this disproportion was considered a crucial difference maker for the impact of the pandemic in these countries (Furlong & Hirshc, 2020).

Let’s take stock. European public health measures and strategies around COVID-19 have predominantly been informed by the biomedical sciences. Other sciences, in particular the social sciences, have hardly contributed to the evidential base of the pandemic crisis management by European governments.^8^ Note that our point is not that other sciences have been *completely* excluded. Economic projections informed cost–benefit analyses and policy-decisions; some of the reports with policy-impact published by the *Leopoldina* in Germany have been authored by expert groups with a minority of biomedical scientists (e.g. Leopoldina, 2020)^9^; and one of the two main French expert groups includes anthropologists, sociologists and ethicists. But our reconstruction of events shows that these and similar examples must be understood against the backdrop of the epistemic hegemony of biomedicine (Sell et al., 2021).

This hegemony is problematic because the COVID-19 pandemic as well as pandemics and epidemics more generally cannot be conceptualised as purely biological phenomena. Rather, pandemics have social and political aspects and consequences. Hence, they need to be considered as “syndemics” (Horton, 2020) with entangled biological and social aspects. This implies that policy-makers need to widen their perspective and take the social aspects of COVID-19 more seriously. Taking these aspects more seriously means taking *scientific evidence and expertise* on social and political phenomena into account properly. This demand suggests a central place for the social sciences as providers of this kind of evidence and expertise at the table of evidence-based policy. To be fair, social aspects will be addressed to some extent in social epidemiology and public health (the research area), e.g. with respect to health effects of social structure.^10^ But the social sciences (in particular sociology, political science, and cultural anthropology) have a broader perspective that includes non-health-related social and political aspects of pandemics. Although there may be alternative ways to address these issues (e.g. expanding social epidemiology), we believe that the social sciences are currently our best available option.^11^

## A role for the social sciences

From an epistemological point of view, the focus on one epistemic perspective (biomedicine) and the lack of disciplinary diversity in policy can be understood as a case of insufficient epistemic pluralism (Lohse & Bschir, 2020). Insufficient epistemic pluralism is problematic because it can limit our options to understand a vastly unknown but policy-relevant reality, which is especially true in light of the fact that scientific knowledge is always perspectival and fallible. But what does a lack of epistemic pluralism actually imply in policy contexts, in particular in relation to the social sciences? To answer this question we will use the COVID-19 pandemic to show that the social sciences can contribute unique perspectives and essential evidence to understanding and managing pandemics. More specifically, we will illustrate three ways in which the social sciences can contribute essential elements to evidence-based pandemic management. We are not the first to bring up these points (e.g., Bavel et al., 2020; Popa, 2021) and we rely on work by others, but it will be useful to list and discuss several examples to show that the social sciences can be important to public health policy *in many more ways* than one might expect.

###  Surveilling

Pandemic surveillance is one of the key functions provided and supported by the sciences during pandemics – a function that could indeed benefit from social scientific input. In particular, the social sciences could improve our ability to understand in which kinds of social situations people are (more) likely to get infected with a highly infectious virus, for instance by systematically backtracking sub-samples (e.g. people with certain occupations or socio-economic backgrounds) of infected people (Streeck, 2021). More globally, the social sciences could help to monitor pandemics and their transmission dynamics. Consider COVID-19 prevalence: the social sciences could have brought critical considerations regarding data and testing strategies to the table, including generating representative samples in different countries to better understand the influence of social structure on disease transmission dynamics – a task that is far from trivial. Many social scientists (and economists) have demanded representative surveys throughout the COVID-19 pandemic, to reduce our ignorance regarding the actual number of infected people and to better assess the effectiveness of different types of policy interventions. A key reason for this demand lies in serious methodological problems in comparing countries with different testing strategies, heterogeneous populations and a different mix of health interventions to estimate the effectiveness of interventions such as school closures and curfews (we will come back to this point).

In addition, social scientific expertise and tools can shed light on several contextual features of data practices, for example by providing information on different testing strategies. This is crucial knowledge because it helps with the comparison of different and local datasets: how can we compare infection rates when some regions of a country have access to more testing units than others and when threshold levels for test results change over time? The social sciences can be key here because contextual factors such as these are frequently latent aspects of data practices and can only partially be accounted for by statistical estimates. For instance, most testing in Europe and elsewhere during the pandemic was recorded locally, with counting practices and testing criteria often differing and changing significantly between municipalities and regions of the same country (Porter, 2021). This, however, was only occasionally apparent to – and rarely fully appreciated by – policy-makers and the media. Rather, data use was often underpinned by the assumption that quantitative data convey reliable and unambiguous information, which can be applied directly to decision-making (Leonelli, 2021a). Social scientific investigations can help to question this assumption and to unearth social dynamics and local discrepancies in data practices – thus crucially improving data interpretation, integration, and harmonisation.

The relevance of the social sciences for pandemic surveillance is even more evident in the context of digital health monitoring tools, such as smartphone apps and fitness trackers. Klingwort and Schnell (2020) draw on survey theory and social scientific findings to show that the use of these digital tools to track the spread of COVID-19 in the population, as suggested by several health agencies, would lead to unreliable conclusions because they will be based on biased samples with unknown population coverage as well as systematic underrepresentation of marginalised groups. Note that this type of criticism is not merely rooted in statistics, but draws on country-specific knowledge regarding social structure and other social aspects, including affinity to digital technology and prevalence of smartphone use.

Surveillance is not only relevant for understanding prevalence and transmission dynamics of infectious diseases in different parts of society, but also to monitor policy interventions and adapt to unanticipated consequences of policy measures. For example, compliance with social distancing rules and mask-wearing can be investigated using social research methods such as interviews and questionnaires (see Munzert & Selb, 2020 for challenges in this context). A (rare) best practice example for this type of surveillance is the Mannheim Corona Study (Blom et al., 2020), which informed the federal government in Germany regarding socio-economic effects of the pandemic and “the influence of political measures on social interactions, fears and the social acceptance of measures to contain the pandemic” based on a daily updated representative longitudinal study.^12^ This type of research can contribute to a more fine grained picture of challenges to adopting health measures in different social groups and, even more importantly, it can generate evidence for more comprehensive harm-benefit analyses. This may include data pointing to indirect social harms such as growing inequalities and social disruption, harms that happen beneath the surface and that may – without a social scientific perspective – stay undetected or at least unrecognised in its true extent (Lohse & Bschir, 2020).

### Predicting

There is extensive literature on prevalent social problems in Europe that is highly relevant for pandemics, including findings on class and milieu inequalities, gender relations, domestic violence, structural racism, insufficient social participation of people with disabilities, and related issues. Social scientific knowledge of this kind is necessary to make predictions regarding likely short- and mid-term societal consequences of pandemics and mitigation policies. This applies because there is a strong correlation between socioeconomic status and health. This includes not only aspects that are common knowledge in epidemiology (i.e. socio-economic differences in vulnerability due to pre-existing conditions), but also social factors that contribute more indirectly to this relationship, for instance through a higher likelihood of the transmission of an infectious respiratory disease in low-income groups with fewer opportunities for reducing job-related mobility, more frequent use of public transportation and grocery shopping in crowded supermarkets, etc.^13^ Furthermore, it is hardly surprising from a sociological perspective that lockdown measures can have vastly different effects on children from different socio-economic backgrounds. Likewise, it would be possible to predict – at least qualitatively^14^ – gender-related differences in childcare during lockdowns that stabilise pre-existing inequalities between men and women (Walter, 2021).

In addition, sociologists, cultural scholars, and political scientists have long addressed specific societal effects of past epidemics, such as the SARS (CoV-1) epidemic (2002–2004, mostly in parts of Asia) and the Western African Ebola Virus epidemic (2014–2016). Consider the edited volume *SARS in China. Prelude to Pandemic?* (Kleinman & Watson, 2006), which investigates the underlying question of what we can learn from the SARS outbreak in China for future pandemics. The book comprises several articles on societal aspects of the epidemic in China, including the political and health system infrastructure underlying China’s response to the epidemic and social stigmatisation issues in the context of the disease. While it is clear that these findings cannot be transferred directly to other pandemics and parts of the world, they may indeed be useful for identifying best practice approaches in health care administration, improve prediction of likely and unlikely scenarios, or at least raise awareness for potential socio-political issues.

A third point concerns the type of epidemiological modelling that, as we have seen, has been in the centre of predicting the spread of the virus and the impact of mitigation measures for COVID-19. Many scholars have emphasised that these models are based on uncertain assumptions and have criticised their role in policy-making for the lack of consideration of these epistemic limitations (Saltelli et al., 2020; Sridhar & Majumder, 2020; see, however, Maziarz & Zach, 2020 and the discussion in van Baßhuysen & White, 2021). What we want to add to the discussion is a potential critical contribution of the social sciences to models and their use for policy. The social sciences could help highlight the multidimensional heterogeneity of different societies, thus avoiding unwarranted conclusions by analogy, based on coarse-grained computer simulations and oversimplifying comparisons between not-so-homologous countries. Furthermore, knowledge about social networks could help modelling different parts of individual societies. Manzo (2020) argues that prevalent epidemiological models neither include information about ‘social hubs’ (individuals with many social contacts) nor about relevant research on contact networks and behavioural patterns, which, however, are highly relevant for modelling disease transmission patterns on a more fine grained level – a level that would most likely be relevant for targeted public health measures (see Herrmann & Schwartz, 2020).^15^ Finally, the social sciences could help to cope with one of the most difficult problems of modelling pandemics: social feedback effects of pandemic-, policy- and prediction-induced human behavioural changes (Friedman et al., 2020; Holmdahl & Buckee, 2020; van Baßhuysen et al., 2021). These can play a decisive role during pandemics, such as when people started to underestimate the dynamic spread of COVID-19 in autumn/winter 2020, partly as a consequence of the abatement of the first wave and relatively unproblematic summer months, which in turn were at least in part a result of behavioural change encouraged by epidemiological projections.

### Intervening

The social sciences can also contribute to increasing the effectiveness of public health measures. In general, social scientific findings on designing choice architectures, communication of policy, and public compliance are an important asset for public health policy-making, as for instance non-compliance regarding self-isolation in quarantine and using face masks is cited as an important challenge to transmission mitigation (Nofal et al., 2020). Based on the importance of compliance with non-pharmaceutical strategies, Michie et al. (2020) have argued for the claim that “behavioural science must be at the heart of the public health response” to the COVID-19 pandemic. A key reason for this claim ties in with the observation made by several philosophers of science and policy (e.g. Cartwright & Stegenga, 2011) that we need to do more than to identify a *potentially* effective mechanism to assess the effectiveness of a policy intervention. We need substantial and detailed knowledge regarding the societal environment of a suggested policy measure to allow for a meaningful assessment. Under what circumstances do curfews work? To what extent are people in the UK, France or Italy less likely to adhere to travel restrictions? In finding answers to questions like these (social) context is king.

Another way in which the social sciences could contribute to the effectiveness of pandemic crisis management is by including knowledge of key stakeholders into policy-making. In many cases, this is done by ad-hoc policy advice by (non-transparently) chosen individuals and organisations (Oliver & Pearce, 2017). Qualitative research and surveys, on the other hand, could contribute to a more systematic inclusion of perspectives and local knowledge.^16^ This includes evidence on the effects of school closures, day-care centres and other important parts of social infrastructure, but also viewpoints that might be relevant for fine-tuning policy measures – such as decisions regarding feasible and manageable hygiene and sanitation in primary schools, informed by experiences and knowledge of people on the ground.

The social sciences can be crucial for devising policy measures that have less (unintended) side-effects and are better targeted. An example for the first part of this claim are mobility restriction approaches that attempt to integrate epidemiological effectiveness and socio-economic feasibility (see the smart mobility concept in the interdisciplinary “No COVID” strategy paper^17^). Or consider the social network modelling by Kaiser et al. (2021) investigating different ways to divide school classes (to reduce the risk of COVID-19 outbreaks in schools) and ways to minimise unintended out-of-school contacts between students as a way to fine-tune lock-down policies. Better *targeted* policy measures could address marginalised communities and vulnerable groups more successfully, by providing information regarding the distribution of face masks in socio-economically disadvantaged groups and by targeted information campaigns that are informed by social research on minorities that may be more likely to respond to non-mainstream communication approaches, among other things. All of these examples represent ways the social sciences could be key in devising proportional pandemic policies with a more local focus instead of using “the hammer” (Pueyo, 2020) all too often.

We take it that our discussion shows that the social sciences could have played a key role in the COVID-19 pandemic and should play a more prominent role in similar public health crises in the future. The social sciences can contribute invaluable insights and make fruitful contributions at various levels to better deal with the effects of pandemics and the unintended side-effects of pandemic management. The social sciences do not only have the capacity to improve existing monitoring/prediction strategies and broaden the evidential basis for policy-making, in particular by providing contextual knowledge. They can also diversify the available perspectives on a public health issue and *ipso facto* expand the options for public health policy measures.

## Epistemic challenges for including the social sciences

If our assessment is correct, we should strive for a tighter integration of the social sciences into evidence-based policy-making. We should also ask why the social sciences were not more involved in COVID-19 crisis management and, more importantly, to what extent (and how) it may be possible to change the epistemic status quo to be better prepared for the next pandemic. However, as we move towards a more pluralistic evidential basis for health policy, challenges and inhibiting factors can be expected at various levels. As a first step to address these, we identify and discuss several *epistemic* challenges below.

Before we continue, we need to make four remarks to help framing our discussion and its underlying goals. (1) Some of the challenges for knowledge integration we want to discuss are specific to the public health context, some are more general and well-known in philosophy of science. Nevertheless, it is useful to highlight the latter’s importance in *this* context. (2) Several issues have a long and complex history, to which we will not be able to do justice within the scope of this paper. Then again, this is not required for the systematic purposes of our discussion. (3) Many of the epistemic challenges that we want to discuss overlap and interact in interesting ways. For this reason, the next passages should be understood as an analytical description of a more complex and more entangled reality. (4) We believe that in reality some combination of epistemic challenges *and sociological factors* has inhibited a more prominent role of the social sciences during the COVID-19 pandemic. Sociological factors may include differences in public prestige between biomedicine and the social sciences, institutional regimes that prioritise certain forms of knowledge, and different styles of public and media engagement of “scientists offering facts” and “public intellectuals providing critical reflections on policy” (cf. Busch, 2009). However, this paper does not aim at a historical *explanation* (including sociological elements) for the lack of social scientific expertise in policy-making during the COVID-19 pandemic. We believe nevertheless that our discussion of epistemic *c*hallenges for a tighter integration of the social sciences in public health policy can be useful. For one thing, it provides a starting point for an actual historical explanation of events in 2020–2021. Furthermore, it can help us think critically about potential obstacles – and ways to overcome these – on our way to pluralise evidence-based public health policy. Against this backdrop, we focus on epistemic aspects, organising our discussion into conceptual, epistemological and methodological challenges.

### Conceptual challenges

The concepts ‘disease’ and ‘public health’, as they are used in public discourse today, are biomedicine-centric concepts. This does not mean that no one is aware that diseases have psycho-social components or that public health also depends on socio-economic factors. But the biomedical perspective is the *dominant* perspective defining the use and connotation of these concepts. Diseases (with the notable exception of psychiatric syndromes) are primarily seen as biological phenomena that are caused by biological and other material factors, such as viruses, toxicological substances and unhealthy diets. In public health, there is a similar tendency to highlight biological aspects of factors that influence the health of the population. As several philosophers and sociologists of medicine and public health scholars have highlighted in recent years, this tendency is usually coupled with a more general disregard for socio-economic causes of disease in epidemiology and biomedical research (Clarke et al., 2019; Hinchliffe et al., 2018; Marmot, 2005). According to Sean Valles (2018, 2019), this disregard is connected to a methodological and epistemological insistence on identifying single, central, and specific causes of disease: in this sense virus infections have the causal specificity that social factors such as education lack (Lloyd, 2002). In epidemiology, social aspects are admittedly frequently taken into account when investigating the determinants of health and disease, especially in social epidemiology (Broadbent, 2013). Yet these are still disregarded as proper causal factors – they are often reduced to mere indicators or background conditions – and the interactions between social factors, biological processes, and health are (still) largely unclear (Parkkinen et al., 2018; Ghiara & Russo, 2019; Kelly & Russo, 2021). As Sheila Jasanoff puts it in a recent interview on the pandemic:

“Attention to physical and material causes is always more highly valued than attention to social causes and consequences. Social sciences and social problems get lower billing and lesser attention than physical and material causes that we think we can control more easily” (Arjini, 2020).

This has immediate consequences for factors that are deemed relevant for the representation and prediction of pandemics and their public health consequences, and we should thus not be surprised that social aspects and social scientific expertise have played a small role throughout the COVID-19 pandemic.

There are also more indirect ramifications of the conceptual dominance of biomedicine for evidence-based public health policy. As Bacevic (2020) has pointed out, the types of knowledge and expertise that form the evidential basis for policy-making are determined by the types of questions policy-makers do and *do not* ask (also see Bacevic & McGoey, 2021). On the one hand, this depends on political views and priorities, of course. On the other hand, the kinds of questions policy-makers ask also depend on their understanding of the extension of ‘public health’ and relevant facets of this concept. If the social dimension of the concept of public health is marginalised, we should not expect policy-makers to heavily draw on social scientific expertise in dealing with the next pandemic.

Hence, conceptual questions are highly relevant in this context and we should critically discuss and possibly conceptually redesign our concept of ‘public health’ (along the lines of an ameliorative explication, see Dutilh Novaes, 2020). Philosophers, scientists and policy-makers should, in other words, ask again what the extension of this concept *should* be, how it relates to the broader concept of public welfare, and what types of causal factors (biological, social, economic etc.) should be considered when policy-makers think about public health threats and appropriate mitigation policies. Finding new answers to these and related questions would be an important step in our way forward to a more pluralistic approach to public health policy.

### Epistemological challenges

Whenever scientific disciplines come together in a multi-, inter- or transdisciplinary setting, the significance of disciplinary boundaries becomes highly visible. There is a large body of work investigating social dynamics and “political” issues in those settings, including work on different epistemic cultures (especially in life and social sciences), competition between epistemic communities, lack of appreciation for other disciplines (and in turn lack of mutual engagement, trust and understanding), disciplinary gate keeping, etc. (e.g. Albert et al., 2009, 2015; Brewer, 1999; O’Malley, 2013). In addition to these issues, there are epistemological challenges that affect the integration of the social sciences into public health policy. Biomedical and social scientists do not only use different languages, conceptual frameworks, and methodologies: the *types* of knowledge available and aimed for in the biomedical and social sciences are extremely different.

First, much of the available knowledge that exists in the social sciences is not as quantified and general as biomedical knowledge. Although there is quantitative research in the social sciences that aims at generalisability, a substantial part of social scientific knowledge is decidedly non-quantitative. This does not only concern sociological and (descriptive) political theory, which is often (but not always) expressed qualitatively. In addition, much empirical research in the social sciences is qualitative in nature, focusing on a rich description of a particular social situation, group or episode. This includes ethnographic studies, narrative interview studies and work based on the interpretation of documents and other human artefacts. In all of these cases, evidence is context-specific and data is only occasionally quantified (if at all).^18^ This is clearly in contrast to fields such as epidemiology and virology, where knowledge is in most cases presented quantitatively – including numerically expressed confidence intervals regarding observational data or model projections – and aims for generalisability.

A second epistemological challenge for integrating social science knowledge into public health policy points to another basic difference in knowledge. With respect to many issues and foundational questions, there exists no stable consensus within the social sciences. This does not only hold for theories, methodologies, and empirical claims but runs even deeper:

“[…] in the social sciences, agreed criteria for evaluating evidential quality are as far away as ever. Concepts are contested, and theoretical controversy generally welcomed as an indication of intellectual health. The notion of professional consensus is foreign to the practice of social science” (Young et al., 2002, p. 223).

The social sciences are, in other words, thoroughly multiparadigmatic disciplines. There is no mainstream school or paradigm dominating sociology, political science or cultural anthropology. On the contrary, there are deep frictions and long-lasting foundational controversies within all of these and neighbouring social sciences (Lohse, 2017; Tang, 2011). For instance, in sociology there are rational choice approaches, systems, network and practice theories, and many more schools, paradigms and sub-paradigms. Many of these have divergent – sometimes even incommensurable – epistemic goals (e.g. prediction vs. sense-making), epistemological stances (e.g. interpretationist vs. naturalist), basic ontologies (e.g. individualism vs. holism) and methodological preferences (for certain types of qualitative or quantitative study designs). This is in contrast with the biomedical sciences, which are not as heterogenous and fragmented as the social sciences.^19^ To be sure, there are many empirical controversies in biomedicine (consider different and changing claims on the transmissibility of SARS-Cov-2) as well as a fair share of epistemological and ontological disputes, for example concerning the structure of predictive models in toxicological research and basic properties of genes and viruses (see, e.g., Calabrese & Baldwin, 2003; Stotz et al., 2004; Dupré & Guttinger, 2016).^20^ However, biomedicine is not characterised by the same multitude of deep-cutting and (frequently paralysing) disputes concerning epistemic aims and the legitimacy of different methodological approaches as the social sciences (Kneer & Moebius, 2010). Different approaches in biomedicine share a larger set of epistemic background commitments than social science paradigms, including an endorsement of quantitative and experimental approaches to biomedical problems and phenomena.

These observations are highly relevant for evidence-based policy. Let us assume we want to include more social science expertise on societal inequalities in public health policy. Who should policy-makers ask? We should expect huge differences regarding the root causes of inequality in Europe and ways to address these in a pandemic, depending on who we ask – a rational choice theorist or a Neo-Marxist, a qualitative or a quantitative researcher. If we want to address this issue, we need to think about ways to incorporate a plurality of social science perspectives regarding epistemic claims relevant for public health policy. This may be done via pluralistic task forces and by channelling policy-advice through professional associations that have an overview of the multiparadigmatic landscape of the social sciences. This is a complicated task with institutional challenges of its own – but there is more. We would also need to think about ways to integrate qualitative evidence with quantified knowledge and models in biomedicine (see El-Sayed & Prainsack, 2021). How can and should we weigh and amalgamate these different types of knowledge drawing on different epistemological stances in practice? Policy-makers could, of course, take their cue from evidence-based medicine and opt for a strict evidence hierarchy that prioritises quantitative over qualitative approaches. However, this would structurally disadvantage the social sciences and in particular social science paradigms that are more aligned with qualitative methodologies than with quantitative study designs. Hence, scientists and policy-makers interested in genuine pluralism should explore new ways of translation and evidence integration, for example by training scholars that are versed in social and biomedical languages and practices and may act as facilitators for interdisciplinary exchange. The alternative would mean to favour a *certain way of doing science* under the banner of objectivity (Mercuri, 2020).

### Methodological challenges

Finally, we want to consider methodological challenges for a tighter integration of the social sciences in public health policy. While many of the aforementioned issues at the conceptual and epistemological level persist, there is a distinct set of challenges that pertain to the ways in which research is actually conducted and applied in the social and biomedical sciences – in particular when it comes to data practices.

A first aspect that we want to discuss concerns data selection. As we suggested in the previous section, the social sciences can be crucial at pointing data collection in a more specific direction, by recommending to test certain social groups, at certain points in time, in certain regions, etc. At the same time, however, there are open questions regarding which social aspects would need to be measured in the context of a broader public health framing, how these should be measured, and whether they can be measured in a valid and reliable way. For example, can movements of individuals and interactions within communities be tracked in the context of pandemics? Social scientific expertise and evidence on social interactions within specific communities could be used to develop policies on this and advice on pertinent questions, including whether this is something that *should* be done and on which characteristics of a community an answer to this question might depend. In this sense, the COVID-19 pandemic has already posed significant questions and exacerbated discussions on data at the intersection of issues of surveillance, policing, and monitoring (Kitchin, 2020). With the increasing importance of trends such as personalised medicine and the ‘datification’ of individual practices, the integration of social scientific evidence and considerations is important to provide grounding and trustworthiness to data selection. For instance, more personal and invasive technologies such as wearable devices have been used to track and collect data that can be used to analyse and (possibly) predict COVID-19 infections before symptoms occur (Mishra et al., 2020). This type of data could be used as evidence for policy interventions, such as the early isolation of (possibly) infected individuals, but these considerations would benefit from coordination with social scientific (and ethical) expertise on the sensitivity of these data, their quality and reliability, and suitability for interventions at the social level.^21^ Yet so far the use of these data has mostly been discussed in the biomedical and technology sector, with little interactions with the social sciences and consideration of the contextual and qualitative knowledge that would improve data assessment, integration, and use in coordination with existing evidence and policy (El-Sayed & Prainsack, 2021; Vayena, 2021).

An additional cluster of challenges that we want to highlight concerns practices of data collection and data infrastructure. Evidential standards can differ even on what counts as data – and in particular high-quality data – between the biomedical and social sciences. Epidemiologists often prefer to use data about biological processes in populations and environments as proxies for social processes, over the use of data collected in the social sciences (see e.g. Canali, 2020). The ways in which health and disease are measured and studied in epidemiology are based on individuals as the main units of analysis (which are then aggregated in populations), and this is in clear opposition to data collection practices in several areas of the social sciences (Kelly & Russo, 2021). In addition, the types of data collected in the biomedical sciences often require extensive work before they can be used as biomedical evidence, which makes data practices – including curation, preparation, clustering and ordering – crucial epistemic gateways (Leonelli, 2019a). In contrast, in the social sciences, where a similar amount of work is required to make data traceable and usable as evidence in more than one context, curation practices are not as advanced and archival work is mostly not considered as important. While contextual information, metadata, and the initial raw materials play a crucial role in various areas of biology and medicine, they are not seen as equally important in the social sciences (Boumans & Leonelli, 2020) (although there are signs of change in this regard, see, e.g., plans for a centralised multidisciplinary data infrastructure in Germany^22^). This is also reflected in differences between databases and archives in the biomedical and social sciences: they are much more abundant and central in biomedicine, where they serve as epistemic and institutional grounding for several research communities (Ankeny & Leonelli, 2016).

Thus, for a tighter integration of the social sciences into public health policy, we would need to address issues on the level of data policies, practices, and infrastructure. A long-term goal could be to combine social scientific and biomedical evidence in integrated assessment models for pandemics. Integrated assessment models attempt to combine aggregated evidence from different academic fields, in particular economics and life/earth sciences, to project possible developments (or ‘scenarios’) depending on different policy options and feedback effects. These models have been predominantly used to inform decision-makers in the context of climate policy, but there have also been first steps towards using these in the context of the COVID-19 pandemic (see Raboisson & Lhermie, 2020 and the model by Dorn et al., 2020, integrating economic and biomedical assumptions and data). However, exploring integrated assessment modelling to inform public health policy in times of crisis would mean that we need to address additional methodological problems. Which aspects of biomedical and societal reality do we want to map (this question also has a conceptual dimension, see above)? In which ways can we aggregate and balance different forms of data and uncertainties? How should we deal with social feedback effects and modelling trade-offs (such as between precision and generality)? These and similar issues touch on methodological issues in model building and their value-ladeness and are well-known in discussions on climate modelling and policy-making (Beck & Krueger, 2016). They would also need to be addressed in the context of evidence-based public health policy.

## Conclusions: Normative implications and ways forward

In this paper, we have used the case of the COVID-19 pandemic in Europe to address the question of what kind of knowledge we should incorporate into public health policy. We have shown that policy-making in Europe during the COVID-19 pandemic has been biomedicine-centric in that its evidential basis marginalised input from non-biomedical disciplines. We have then argued that in particular the social sciences could contribute essential expertise and evidence to public health policy in times of biomedical emergencies and that we should thus strive for a tighter integration of the social sciences in future evidence-based policy-making. This demand faces a number of challenges on different levels, which we have identified and discussed as potential inhibitors for a more pluralistic evidential basis. Several challenges at conceptual, epistemological and methodological levels need to be addressed by scientists, policy-makers and philosophers of science, among other interest parties, if we want to improve future public health policy, in particular in times of crisis.

As we have seen, epistemic challenges for a tighter integration of the social sciences in public health policy are manifold and do not give hope for easy solutions. They need to be addressed on different levels and often negotiated between different stakeholder groups. Conceptual decisions about which social aspects should be considered as relevant for a re-engineered concept of public health must be made at the interface of science and policy. Epistemological and methodological discussions regarding data integration and the weighting of evidence will need to involve biomedical and social scientists and possibly also experts on science-based policy. In the course of these discussions, emerging organisational questions will need to be addressed as well. For instance, we would need to think not only about what needs to be measured and how, but about who should be responsible for collecting and storing data on potentially public health-relevant social aspects. Research policy could fund comprehensive programmes to incentivise appropriate social research projects, or the disciplinary horizon of public health agencies could be broadened to include more social scientists and increase the collection of relevant social data. It is likely that approaches will vary across Europe which makes it vital to also think about scientific institutions and data infrastructure in this context. We would need to discuss how we can develop better organised data repositories for the social sciences, to what extent these can be open access (in view of highly sensitive data sets), and, finally, who should be responsible for creating and curating these. Philosophers of science and STS scholars working on big data, data infrastructure, and open science can contribute to these discussions (e.g. Holmberget al., 2013; Leonelli, 2019b; Vayena, 2021).

As a last step, we want to turn a spotlight on the ethical dimension of our epistemic analysis and touch on related points. We first want to stress that thinking about ways to improve future public health policies is not only laudable but also ethically required, considering that the likelihood of new pandemics is higher than ever. Despite the temptation to think that the COVID-19 pandemic was a once-in-a-lifetime event, the emergence of (more) zoonotic diseases and pandemics is unfortunately on the horizon due to the ongoing transformation of ecosystems into agricultural systems and mass animal farming, among other things (Birch, 2021; Gibb et al., 2020). In other words, we might live in a new age of pandemics. As a consequence, we should, of course, strive to limit or discontinue practices that are major anthropogenic drivers for the emergence of new zoonotic diseases, in particular wildlife consumption and animal farming (Wiebers & Feigin, 2020). However, we should also prepare for a future where our efforts in this direction may be of limited success – which seems to be prudent given the lack of appropriate action taken to avoid global warming in the last decades. Preparing for such a future implies thinking critically about ways to improve our public health response to future pandemics and to include essential types of evidence and expertise that the sciences *– including the social sciences* – can provide.

To be clear, the social sciences are important beyond their contribution to policy in terms of evidence and expertise. The critical and reflexive potential of the social sciences exceeds their possible role *within* evidence-based policy. For example, social scientific work that has highlighted the neglect of local expertise and insufficient attention to social issues has been crucial in identifying blind spots of policy. On a more general level, critical social sciences will be important to understand, evaluate and debunk claims of scientific objectivity and evidence-based public health policy-making – including its use of scientific language to conceal political value judgements and its overreliance on the (questionable) objectivity of numbers in justifying policy decisions (see Bogner & Menz, 2021 for an interesting discussion on expertise during the COVID-19 pandemic).^23^ As we have mentioned at the beginning of our analysis, several governments have framed their policy as a way of “following the science” and doing what scientists and evidence told them to do. The social science can provide crucial tools to scrutinise these and similar statements and bring to light the values that actually guide policy-making and its use of scientific evidence. It goes without saying that the humanities, in particular history and philosophy, should be considered important allies in this task.

But even as active contributors to evidence-based policy, the social sciences are much more than providers of evidence. They can add an independent perspective and open up additional dimensions in our understanding of pandemics. This is an essential aspect of our demand to improve interdisciplinary knowledge integration in public health policy. Without a more pluralistic body of evidence, certain issues will be addressed disproportionately in policy-making and in particular social aspects will be underrepresented, eventually leading to myopic goal-setting and imbalanced decision-making in public health policy (which has been a major point of public critique in many European countries). Our point here is not that we would necessarily have had less restrictive COVID-19 policies if we had properly considered “the social side” of the situation in public health policy. Rather, we want to argue that a tighter integration of the social sciences in public health policy would have allowed for better informed and targeted policy measures during the COVID-19 pandemic and will allow for better policy in future public health crises. In particular, it will enable policy-makers to evaluate different policy options on the basis of truly pluralistic evidence that allows for a more adequate discussion of ethical key questions for public health programmes: How effective will this policy measure be in achieving its goals? What are its known burdens to different communities and how can these be minimised or balanced? Are there alternative options that could be implemented?^24^

Addressing these critical questions by including social scientific perspectives can allow for more fine-grained harm-benefit-analyses, and it can help to develop policy options that would be based not only on biomedical data, epidemiological projections and the occasional “expert statement” by social scientists, but also on actual evidence on social issues. Moreover, policies informed by pluralistic and interdisciplinary evidence can, with good reason, be considered to be more objective. There have been several calls for objective evidence and policy in the COVID-19 context, and yet these considerations tend to be restricted to procedural notions of objectivity that consider the use of evidence as a neutral activity and only specific types of data as evidence (Jukola & Canali, 2021). In contrast to these views, incorporating interdisciplinary knowledge can strengthen objectivity by bringing a diversity of approaches and perspectives into contact, thereby avoiding one-sided and potentially biased views of reality (Longino, 1990; see also Mill, 2015[1859], chap. II). Hence, pluralism and interdisciplinary knowledge integration in evidence-based public health policy is justified on ethical and on epistemological grounds.

## Data Availability

Not applicable.

## Footnotes

1 Here and throughout the paper, we use a broad notion of ‘biomedicine’, including epidemiology, life science research with health applications, clinical research, and related health sciences.

2 Our analysis is limited to the period up to the beginning of 2021. It is also limited by the fact that we are writing this as a process continues to unfold, based in part on newspaper articles and preprints, with new information on pandemic policy in Europe emerging on a weekly basis.

3 As a result of our focus on these three stages here, we will not include post-pandemic policy in our analysis.

4 See “Timeline of EU Action” https://ec.europa.eu/info/live-work-travel-eu/coronavirus-response/timeline-eu-action_en (accessed July 2021).

5 The official composition of this expert committee was officially announced in late April and was updated in early 2021. European governments have reveiled their choices of experts in different ways: in some countries, the lists of participants were announced from the start, while in others they have not been officially announced (yet).

6 We use the term ‘lockdown’ with caution here, as it has been used throughout the pandemic to refer to several different and significantly diverse policies, from the closure of non-essential shops to restrictions to travelling and visits to friends and family (including curfews).

7 See “Wie wir COVID-19 unter Kontrolle bekommen” (“How we get COVID-19 under control”), https://www.bmi.bund.de/SharedDocs/downloads/DE/veroeffentlichungen/2020/corona/szenarienpapier-covid19.html (accessed July 2021).

8 As far as we know this is true of other parts of the world too.

9 The German National Academy of Sciences *Leopoldina* is a historic academic institution, similar to the Royal Society in the UK, whose role includes the representation of the German scientific community and the development of science-based advice for policy-making and the public.

10 See, e.g., the policy briefings by the *Competence Network for Public Health COVID-19,* https://www.public-health-covid19.de/en/results.html (accessed July 2021).

11 Note that social epidemiology and other fields that overlap with social science research have been largely left out of the policy response to the COVID-19 pandemic too.

12 See https://www.uni-mannheim.de/en/gip/corona-study/ and https://www.uni-mannheim.de/newsroom/presse/pressemitteilungen/2020/april/corona-studie-teil-4/ (accessed July 2021). See also Germany’s COSMO snap shot monitoring, https://projekte.uni-erfurt.de/cosmo2020/web/ (accessed July 2021).

13 Chang et al.’s (2021) mobility network study, which combines biomedical and social scientific expertise with sophisticated informatics to better understand demographic and socio-economic differences in COVID-19 infections, vividly illustrates the relevance of these factors for the pandemic.

14 This qualification reflects the hitherto lack of success in quantitatively exact predictions in the social sciences.

15 Note that social network modelling is not only a research field in the social sciences, but is frequently truly interdisciplinary, integrating work from the social, life, physical and computer sciences.

16 There is also an argument to made for a more direct inclusion of local knowledge, external to science, in evidence-based public health policy (Bschir & Lohse, 2021; Kearnes et al., 2020).

17 https://www.ifo.de/DocDL/Fuest_etal_2021_proaktive_Bekaempfung_SARS-CoV-2_Handlungsoptionen.pdf (accessed February 2021), see also Horton (2021).

18 The question of the extent to which findings of qualitative studies can – or should – be expressed quantitatively is a thorny issue in the (philosophy of the) social sciences. If interpretationists are right in claiming that this is *in principle* not viable, this would point to even bigger epistemological incompatibilities between social and biomedical sciences (see also the debate on reasons vs. causes D’Oro & Sandis, 2013). However, in this paper we will only make the weaker claim that much social scientific knowledge is indeed qualitative and can – for pragmatic reasons – hardly be comprehensively transformed into quantitative knowledge.

19 Note that economics has a dominating (although not completely uncontested) paradigm and is to a much higher degree mathematised than the (other) social sciences (Lohse, 2017), which arguably makes it more compatible and easier to integrate with biomedical research.

20 We are indebted to an anonymous reviewer for pointing us towards these examples and the non-empirical nature of many biomedical controversies.

21 Sabina Leonelli (2021b) makes a compelling case for including community representatives alongside social scientists in decisions on these issues to enhance normative awareness and increase benefits for both researchers and the public.

22 See https://www.dfg.de/en/research_funding/programmes/nfdi/index.html (accessed July 2021).

23 At the same time, we think that the interdisciplinary and pluralistic direction we suggest in the article can avoid the risk of sometimes oversimplifying "anti-science critiques" that have been advanced. Discussions that include both biomedical scientists and social scientists can help to provide more complex answers to difficult public health questions – rather than merely highlighting the limitations of one side of the discussion.

24 See the ethical framework for public health programmes developed by Kass (2001) for a detailed discussion of these and related questions.

## References

[CR1] Adam D (2020). Special report: The simulations driving the world’s response to COVID-19. Nature.

[CR2] Albert M, Laberge S, Hodges BD (2009). Boundary-work in the health research field: Biomedical and clinician scientists’ perceptions of social science research. Minerva.

[CR3] Albert M, Paradis E, Kuper A (2015). Interdisciplinary promises versus practices in medicine: The decoupled experiences of social sciences and humanities scholars. Social Science & Medicine.

[CR4] Allen, D., Stanczyk, L., Sethi, R., & Weyl, G. (2020). *When can we go out? Evaluating policy paradigms for responding to the COVID-19 threat*. https://ethics.harvard.edu/when-can-we-go-out

[CR5] Ankeny RA, Leonelli S (2016). Repertoires: A post-Kuhnian perspective on scientific change and collaborative research. Studies in History and Philosophy of Science Part A.

[CR6] Arjini, N. (2020, 6 April). Science will not come on a white horse with a solution (Interview with Sheila Jasanoff). *The Nation*. https://www.thenation.com/article/society/sheila-jasanoff-interview-coronavirus/

[CR7] Bacevic, J., & McGoey, L. (2021). Surfing ignorance: Covid-19 and the rise of fatalistic liberalism. *SocArXiv*. 10.31235/osf.io/pxju7

[CR8] Bacevic, J. (2020, 28 April). There’s no such thing as just ‘following the science’ – advice is political. *The Guardian*. https://www.theguardian.com/commentisfree/2020/apr/28/theres-no-such-thing-just-following-the-science-coronavirus-advice-political

[CR9] Bavel, J. J. V., Baicker, K., Boggio, P. S., Capraro, V., Cichocka, A., Cikara, M., Crockett, M. J., Crum, A. J., Douglas, K. M., Druckman, J. N., Drury, J., Dube, O., Ellemers, N., Finkel, E. J., Fowler, J. H., Gelfand, M., Han, S., Haslam, S. A., Jetten, J., …, & Willer, R. (2020). Using social and behavioural science to support COVID-19 pandemic response. *Nature Human Behaviour*, *4*(5), 460–471. 10.1038/s41562-020-0884-z 10.1038/s41562-020-0884-z 32355299

[CR10] Beck M, Krueger T (2016). The epistemic, ethical, and political dimensions of uncertainty in integrated assessment modeling. Wires Climate Change.

[CR11] Birch, J. (2021). Animals, humans and pandemics: What needs to change? *Philosophy, Logic and Scientific Method Blog*. https://www.lse.ac.uk/philosophy/blog/2021/03/09/animals-humans-and-pandemics-what-needs-to-change/

[CR12] Blom, A. G., Wenz, A., Rettig, T., Reifenscheid, M., Naumann, E., Möhring, K., Lehrer, R., Krieger, U., Juhl, S., Friedel, S., Fikel, M., & Cornesse, C. (2020). *The Mannheim Corona study: Life in Germany in a state of emergency : Report for March 20 to July 09, 2020* [working paper]. https://madoc.bib.uni-mannheim.de/55629

[CR13] Bogner, A., & Menz, W. (2021). Wissen und Werte im Widerstreit. Zum Verhältnis von Expertise und Politik in der Corona-Krise. *Leviathan*, *49*(1), 111–132. 10.5771/0340-0425-2021-1-111

[CR14] Boumans M, Leonelli S, Leonelli S, Tempini N (2020). From dirty data to tidy facts: Clustering practices in plant phenomics and business cycle analysis. Data journeys in the sciences.

[CR15] Brewer GD (1999). The challenges of interdisciplinarity. Policy Sciences.

[CR16] Broadbent A (2013). Philosophy of epidemiology.

[CR17] Bschir, K., & Lohse, S. (2021). *Pandemics, policy, and pluralism. A feyerabend-inspired perspective on COVID-19 (unpublished manuscript)*. 10.1007/s11229-022-03923-4 PMC9607765 36320863

[CR18] Busch A (2009). Politikwissenschaft und Politikberatung: Reflektionen anlässlich der aktuellen Krise. Zeitschrift Für Politikberatung.

[CR19] Calabrese EJ, Baldwin LA (2003). Toxicology rethinks its central belief. Nature.

[CR20] Canali, S. (2020). Making evidential claims in epidemiology: Three strategies for the study of the exposome. *Studies in History and Philosophy of Science Part C: Studies in History and Philosophy of Biological and Biomedical Sciences,**82*, 101248. 10.1016/j.shpsc.2019.101248 10.1016/j.shpsc.2019.101248 32307253

[CR21] Cartwright, N., & Hardie, J. (2012). *Evidence-based policy: A practical guide to doing it better*. Oxford University Press.

[CR22] Cartwright N, Stegenga J (2011). Evidence, Inference and Enquiry. Proceedings of the British Academy.

[CR23] Chang S, Pierson E, Koh PW, Gerardin J, Redbird B, Grusky D, Leskovec J (2021). Mobility network models of COVID-19 explain inequities and inform reopening. Nature.

[CR24] Chang, H. (2014). Epistemic activities and systems of practice. Units of analysis in philosophy of science after the practice turn. In L. Soler, S. Zwart, M. Lynch, & V. Israel-Jost (Eds.), *Science after the practice turn in the philosophy, history, and social studies of science* (pp. 67–79). Routledge.

[CR25] Chin V, Ioannidis JPA, Tanner MA, Cripps S (2021). Effect estimates of COVID-19 non-pharmaceutical interventions are non-robust and highly model-dependent. Journal of Clinical Epidemiology.

[CR26] Clarke B, Ghiara V, Russo F (2019). Time to care: Why the humanities and the social sciences belong in the science of health. British Medical Journal Open.

[CR27] D’Oro, G., & Sandis, C. (Eds.) (2013). *Reasons and causes: Causalism and anti-causalism in the philosophy of action*. Palgrave.

[CR28] Devlin, H., & Boseley, S. (2020, 23 April). Scientists criticise UK government’s “following the science” claim. *The Guardian*. https://www.theguardian.com/world/2020/apr/23/scientists-criticise-uk-government-over-following-the-science

[CR29] Dorn, F., Khailaie, S., Stöckli, M., Binder, S., Lange, B., Peichl, A., Vanella, P., Wollmershäuser, T., Fuest, C., & Meyer-Hermann, M. (2020). Das gemeinsame Interesse von Gesundheit und Wirtschaft: Eine Szenarienrechnung zur Eindämmung der Corona- Pandemie. *Ifo Schnelldienst Digital*, *1*(06).

[CR30] Dupré J, Guttinger S (2016). Viruses as living processes. Studies in History and Philosophy of Science Part C: Studies in History and Philosophy of Biological and Biomedical Sciences.

[CR31] Dutilh Novaes C (2020). Carnapian explication and ameliorative analysis: A systematic comparison. Synthese.

[CR32] El-Sayed S, Prainsack B (2021). Blue chips and white collars: Whose data science is it?. Harvard Data Science Review.

[CR33] Ferguson, N.M., Laydon, D., Nedjati-Gilani, G., et al. (2020). Report 9: Impact of non-pharmaceutical interventions (NPIs) to reduce COVID-19 mortality and healthcare demand. 10.25561/77482 10.1007/s11538-020-00726-x PMC7140590 32270376

[CR34] Friedman, J., Liu, P., Troeger, C. E., Carter, A., Reiner, R. C., Barber, R. M., Collins, J., Lim, S. S., Pigott, D. M., Vos, T., Hay, S. I., Murray, C. J. L., & Gakidou, E. (2020). Predictive performance of international COVID-19 mortality forecasting models. *MedRxiv*, 2020.07.13.20151233. 10.1101/2020.07.13.20151233 10.1038/s41467-021-22457-w PMC8110547 33972512

[CR35] Fuller J (2021). What are the COVID-19 models modelling (philosophically speaking)?. History and Philosophy of the Life Sciences.

[CR36] Furlong, A., & Hirshc, C. (2020, 25 March). Charting Europe’s capacity to deal with the coronavirus crisis. *Politico*. https://www.politico.eu/article/charting-europes-capacity-to-deal-with-the-coronavirus-crisis/. Accessed July 2021.

[CR37] Ghiara V, Russo F (2019). Reconstructing the mixed mechanisms of health: The role of bio- and sociomarkers. Longitudinal and Life Course Studies.

[CR38] Gibb R, Redding DW, Chin KQ, Donnelly CA, Blackburn TM, Newbold T, Jones KE (2020). Zoonotic host diversity increases in human-dominated ecosystems. Nature.

[CR39] Goumenou M, Sarigiannis D, Tsatsakis A (2020). COVID-19 in Northern Italy: An integrative overview of factors possibly influencing the sharp increase of the outbreak (Review). Molecular Medicine Reports.

[CR40] Herrmann, H. A., & Schwartz, J.-M. (2020). Why COVID-19 models should incorporate the network of social interactions. *Physical Biology,**17*(6), 065008. 10.1088/1478-3975/aba8ec 10.1088/1478-3975/aba8ec 32702678

[CR41] Hinchliffe S, Jackson MA, Wyatt K, Barlow AE, Barreto M, Clare L, Depledge MH, Durie R, Fleming LE, Groom N, Morrissey K, Salisbury L, Thomas F (2018). Healthy publics: Enabling cultures and environments for health. Palgrave Communications.

[CR42] Holmberg C, Bischof C, Bauer S (2013). Making predictions: Computing populations. Science, Technology, & Human Values.

[CR43] Holmdahl, I., & Buckee, C. (2020). Wrong but Useful—What Covid-19 Epidemiologic Models Can and Cannot Tell Us. *New England Journal of Medicine*, *0*(0), null. 10.1056/NEJMp2016822 10.1056/NEJMp2016822 32412711

[CR44] Horton R (2020). Offline: COVID-19 is not a pandemic. The Lancet.

[CR45] Horton R (2021). Offline: The case for No-COVID. The Lancet.

[CR46] Jasanoff S (1987). Contested boundaries in policy-relevant science. Social Studies of Science.

[CR47] Jasanoff, S., Hilgartner, S., Hurlbut, J. B., Özgöde, O., & Rayzberg, M. (2021). *Comparative covid response: Crisis, knowledge, politics (Interim Report)*. https://www.unicamp.br/unicamp/sites/default/files/2021-01/Harvard-Cornell%20Report%202020.pdf. Accessed Jan 2021.

[CR48] Jukola S, Canali S (2021). On evidence fiascos and judgments in COVID-19 policy. History and Philosophy of the Life Sciences.

[CR49] Kaiser, A., Kretschmer, D., & Leszczensky, L. (2021). Social network-based strategies for classroom size reduction can help limit outbreaks of SARS-CoV-2 in high schools. A simulation study in classrooms of four European countries. *MedRxiv*, 2020.11.30.20241166. 10.1101/2020.11.30.20241166

[CR50] Kass NE (2001). An ethics framework for public health. American Journal of Public Health.

[CR51] Kearnes, M., Cook, B. R., Kuch, D., Leach, J., Stephenson, N., Ankeny, R. A., & Raman, S. (2020, 1 April). We should listen to Coronavirus experts, but local wisdom counts too. *The Conversation*. http://theconversation.com/we-should-listen-to-coronavirus-experts-but-local-wisdom-counts-too-134034

[CR52] Kelly MP, Russo F (2021). The epistemic values at the basis of epidemiology and public health. MEFISTO.

[CR53] Kitchin R (2020). Civil liberties or public health, or civil liberties and public health? Using surveillance technologies to tackle the spread of COVID-19. Space and Polity.

[CR54] Kleinman, A. & Watson, J. L. (2006). *SARS in China: Prelude to pandemic?* Stanford University Press.

[CR55] Klingwort, J., & Schnell, R. (2020). Critical limitations of digital epidemiology: *Survey Research Methods*, *14*(2), 95–101. 10.18148/srm/2020.v14i2.7726

[CR56] Kneer, G., & Moebius, S. (Eds.). (2010). *Soziologische Kontroversen: Beiträge zu einer anderen Geschichte der Wissenschaft vom Sozialen*. Suhrkamp.

[CR57] Leonelli S (2019). What distinguishes data from models?. European Journal for Philosophy of Science.

[CR58] Leonelli S (2019). Data governance is key to interpretation: Reconceptualizing data in data science. Harvard Data Science Review.

[CR59] Leonelli S (2021). Data science in times of pan(dem)ic. Harvard Data Science Review.

[CR60] Leonelli S (2021). Rejoinder: The present and future of data science in society. Harvard Data Science Review.

[CR61] Leopoldina (Nationale Akademie der Wissenschaften). (2020). *Coronavirus-Pandemie – Die Krise nachhaltig überwinden. 3. Ad-hoc-Stellungnahme*: https://www.leopoldina.org/uploads/tx_leopublication/2020_04_13_Coronavirus-Pandemie-Die_Krise_nachhaltig_%C3%BCberwinden_final.pdf. Accessed May 2020.

[CR62] Lloyd EA, Van Regenmortel MHV, Hull DL (2002). Reductionism in medicine: Social aspects of health. Promises and limits of reductionism in the biomedical sciences.

[CR63] Lohse S (2017). Pragmatism, ontology, and philosophy of the social sciences in practice. Philosophy of the Social Sciences.

[CR64] Lohse S, Bschir K (2020). The COVID-19 pandemic: A case for epistemic pluralism in public health policy. History and Philosophy of the Life Sciences.

[CR65] Lohse S, Wasmer M, Reydon T (2020). Integrating philosophy of science into research on ethical, legal and social issues in the life sciences. Perspectives on Science.

[CR66] Longino, H. E. (1990). *Science as Social Knowledge: Values and objectivity in scientific inquiry*. Princeton University Press.

[CR67] Manzo G (2020). Complex social networks are missing in the dominant COVID-19 epidemic models. Sociologica.

[CR68] Marmot M (2005). Social determinants of health inequalities. The Lancet.

[CR69] Maziarz M, Zach M (2020). Agent-based modelling for SARS-CoV-2 epidemic prediction and intervention assessment: A methodological appraisal. Journal of Evaluation in Clinical Practice.

[CR70] Mercuri M (2020). Just follow the science: A government response to a pandemic. Journal of Evaluation in Clinical Practice.

[CR71] Michie, S., Rubin, J., & Amlôt, R. (2020, 28 February). Behavioural science must be at the heart of the public health response to covid-19. *The BMJ*. https://blogs.bmj.com/bmj/2020/02/28/behavioural-science-must-be-at-the-heart-of-the-public-health-response-to-covid-19/

[CR72] Mill JS (2015). On liberty.

[CR73] Mishra T, Wang M, Metwally AA (2020). Pre-symptomatic detection of COVID-19 from smartwatch data. Nature Biomedical Engineering.

[CR74] Morgan MS (2012). Case studies: One observation or many? Justification or discovery?. Philosophy of Science.

[CR75] Munzert, S., & Selb, P. (2020). Can we directly survey adherence to non-pharmaceutical interventions? *Survey Research Methods*, *14*(2), 205–209. 10.18148/srm/2020.v14i2.7759

[CR76] Nofal, A. M., Cacciotti, G., & Lee, N. (2020). Who complies with COVID-19 transmission mitigation behavioral guidelines? *PLoS ONE,**15*(10), e0240396. 10.1371/journal.pone.0240396 10.1371/journal.pone.0240396 PMC7544078 33031476

[CR77] Nutley, S., Morton, S., Jung, T., & Boaz, A. (2010). Evidence and policy in six European countries: Diverse approaches and common challenges. *Evidence & Policy: A Journal of Research, Debate and Practice,**6*(2), 131–144. 10.1332/174426410X502275.

[CR78] O’Malley MA (2013). When integration fails: Prokaryote phylogeny and the tree of life. Studies in History and Philosophy of Science Part C: Studies in History and Philosophy of Biological and Biomedical Sciences.

[CR79] Oliver K, Pearce W (2017). Three lessons from evidence-based medicine and policy: Increase transparency, balance inputs and understand power. Palgrave Communications.

[CR80] Parkkinen V-P, Wallmann C, Wilde M, Clarke B, Illari P, Kelly MP, Norell C, Russo F, Shaw B, Williamson J (2018). Evaluating evidence of mechanisms in medicine: Principles and procedures. Springer International Publishing.

[CR81] Pistoi S (2021). Examining the role of the Italian COVID-19 scientific committee. Nature Italy.

[CR82] Popa E (2021). Loneliness and negative effects on mental health as trade-offs of the policy response to COVID-19. History and Philosophy of the Life Sciences.

[CR83] Porter TM (2021). A plague of data. Harvard Data Science Review.

[CR84] Pueyo, T. (2020). Coronavirus: The hammer and the dance. *Medium*. https://medium.com/@tomaspueyo/coronavirus-the-hammer-and-the-dance-be9337092b56

[CR85] Raboisson, D., & Lhermie, G. (2020). Living with COVID-19: A systemic and multi-criteria approach to enact evidence-based health policy. *Frontiers in Public Health*, *8*. 10.3389/fpubh.2020.00294 10.3389/fpubh.2020.00294 PMC7308759 32612973

[CR86] Remuzzi A, Remuzzi G (2020). COVID-19 and Italy: What next?. The Lancet.

[CR87] Saltelli, A., Bammer, G., Bruno, I., Charters, E., Di Fiore, M., Didier, E., Nelson Espeland, W., Kay, J., Lo Piano, S., Mayo, D., Pielke Jr, R., Portaluri, T., Porter, T. M., Puy, A., Rafols, I., Ravetz, J. R., Reinert, E., Sarewitz, D., Stark, P. B., …, & Vineis, P. (2020). Five ways to ensure that models serve society: A manifesto. *Nature*, *582*(7813), 482–484. 10.1038/d41586-020-01812-9 10.1038/d41586-020-01812-9 32581374

[CR88] Sell, K., Saringer-Hamiti, L., Geffert, K., Strahwald, B., Stratil, J. M., & Pfadenhauer, L. M. (2021). Politikberatung durch Expert*innenräte in der SARS-CoV-2-Pandemie in Deutschland: Eine Dokumentenanalyse aus Public-Health-Perspektive. *Zeitschrift für Evidenz, Fortbildung und Qualität im Gesundheitswesen*. 10.1016/j.zefq.2021.06.002 10.1016/j.zefq.2021.06.002 PMC8404986 34474991

[CR89] Sridhar, D., & Majumder, M. S. (2020). Modelling the pandemic. *BMJ,**369,*. m1567. 10.1136/bmj.m1567 10.1136/bmj.m1567 32317328

[CR90] Stevens, A. (2007). Survival of the ideas that fit: An evolutionary analogy for the use of evidence in policy. *Social Policy and Society,**6*(1), 25–35. 10.1017/S1474746406003319.

[CR91] Stotz K, Griffiths PE, Knight R (2004). How biologists conceptualize genes: An empirical study. Studies in History and Philosophy of Science Part C: Studies in History and Philosophy of Biological and Biomedical Sciences.

[CR92] Streeck, W. (2021, 11 January). Alternativen zum Lockdown: Welchen Wissenschaftlern folgen wir in der Pandemie? *FAZ.NET*. https://www.faz.net/1.7138966

[CR93] Tang S (2011). Foundational Paradigms of Social Sciences. Philosophy of the Social Sciences.

[CR94] Valles SA (2019). A pluralistic and socially responsible philosophy of epidemiology field should actively engage with social determinants of health and health disparities. Synthese.

[CR95] Valles, S. A. (2018). *Philosophy of Population Health: Philosophy for a New Public Health Era*. Routledge.

[CR96] van Baßhuysen, P., & White, L. (2021). *How philosophers of science violated their epistemic duties during the SARS-CoV-2 crisis*. http://philsci-archive.pitt.edu/18584/1/2021%20Draft%20Paper%20How%20Philosophers%20of%20Science%20Violated%20Their%20Epistemic%20Duties%20During%20the%20SARS-CoV-2%20Crisis.pdf. Accessed May 2021.

[CR97] van Baßhuysen, P., White, L., Khosrowi, D., & Frisch, M. (2021). Three ways in which pandemic models may perform a pandemic. *Erasmus Journal for Philosophy and Economics*, *14*(1). 10.23941/ejpe.v14i1.582

[CR98] Vayena E (2021). Value from health data: European opportunity to catalyse progress in digital health. The Lancet.

[CR99] Walter, N. (2021, 28 February). *Guilt and fury: How Covid brought mothers to breaking point. **The Guardian**. *http://www.theguardian.com/lifeandstyle/2021/feb/28/mums-women-coronavirus-covid-home-schooling-inequality

[CR100] Wiebers DO, Feigin VL (2020). What the COVID-19 crisis is telling humanity. Neuroepidemiology.

[CR101] Young K, Ashby D, Boaz A, Grayson L (2002). Social science and the evidence-based policy movement. Social Policy and Society.

